# Hypodermic Injection in a Case of Inertia from Exhaustion

**Published:** 1869-07

**Authors:** T. G. Williams

**Affiliations:** Watertown, Wisconsin


ARTICLE XXVII.

### HYPODERMIC INJECTION IN A CASE OF INERTIA FROM EXHAUSTION.

BY. T. G. WILLIAMS, Watertown, Wisconsin.

DEAR EXAMINER:—If you think the following case of sufficient importance, you may record it.

Mrs. J., age 43, mother of several children, passed through her previous confinement naturally and easy. During her last gestation, she had been debilitated greatly, on account of a family trouble. She thinks that she passed beyond the usual time before the labor commenced. *May 9th.* Labor apparently commenced; and an intelligent midwife was in attendance. After two days, not seeing much progress in the case, the midwife left, with instructions that she be called when necessary. Patient continued a week having troublesome pains at night, with none, scarcely, during the day. During this time she obtained but little sleep. *May 16th: 10 A.M.* Membranes ruptured; an immense quantity of liquor amnii passing off. Powerful pains set in after this, but with no results further than to exhaust the patient. Saw her, 9 P.M., same day, exhausted, having but slight pains. Found right arm down, breech and head to patient's right, and right side of fœtus to patient's left. Fœtal heart seemed weak. Introduced hand for turning. Os well dilated. Found uterus distensible, and fœtus somewhat movable. Owing to inertia, I felt certain that child could not be delivered rapidly enough by the breech, and live, so I tried bringing head down. Returned the arm, and, by pressing shoulder up, succeeded in bringing head down into 3d position; occiput to right acetabulum. Pains increased sufficient to keep it in position, but did not propel it onward in the least. Stimulation seemed only to increase her suffering. No rest possible, owing to pain. Waited from 9.30 P.M. to 1 A.M., and no advance. Patient weak and very restless. Gave hypodermic inj.—morph. sulph, gr. $\frac{1}{6}$. In ten minutes, patient was quiet, and slept considerable during the next half hour. 40 minutes after the inj. was given, pains commenced, and in 20 minutes more, a child weighing 9½ ℔s was born. Head more ossified than natural. Mother did well. No hemorrhage. Two turns of cord drawn tightly about the neck of child, and pulsation nearly ceased. Artificial respiration and other efforts succeeded in restoring life after 30 minutes. During this time it took but one or two gasps of its own accord, and heart action was very weak at times. Both doing well.

